# Calamities of Surgery

**Published:** 1876-07

**Authors:** 


### Our Exchanges.

---

"Calamities of Surgery."—This is the title of one of the very valuable series of "Clinical Lectures and Essays" recently published by Sir James Paget. It contains so many hints for the direction of those who devote themselves to the subject of surgery that a brief synopsis of it will not be unprofitable.

The author treats the subject under two leading heads; first, the care that the surgeon should exercise in deciding upon and recommending an operation; and secondly, the various necessary precautions that should be observed after an operation has been decided upon.

Under the first division of the subject, the author speaks as follows: "First of all, the consideration that you are liable to these calamities should be an incentive to the most earnest and continued study of your profession, that you may avert all avoidable ignorance; and to constant discipline in watchfulness, that you may overlook nothing that can contribute to a patient's welfare.

"And you should study very carefully all of what are called the minor points of your profession. . . . I refer chiefly to the necessity of cultivating skill in dressing wounds, in the completion of operations, in the looking to the seemingly little things that, after an operation, minister not only to a patient's comfort, but to his welfare.

"Next, let the liability to these calamities, move you never to decide upon an operation except in consideration of the patient's interests alone." This is a warning which, it is to be feared, is not always as conscientiously followed as it ought to be. Rising surgeons, in their desire to acquire a reputation in their professlon, too often undertake operations of doubtful propriety, from the fact that the issue of such operations is at best only problematical. On this point Sir James Paget very judiciously says: "When an operation is decided on, you may add a desire for your own reputation to the motives that will induce you to do the best you possibly can for the patient; but this, which is a very fair motive for the careful performance of an operation, is a very foul one in determining whether an operation should be done or not."

The next item of useful advice which the distinguished author presents is, "never to decide upon an operation, even of a trivial kind, without first examining the patient as to the risks of his life. You should examine him with at least as much care as you would for a life insurance. It is surely at least as important that a man should not die or suffer serious damage after an operation, as that his life should be safely insured for a few hundred pounds." After dwelling upon the importance of observing this precaution at considerable length, Sir James Paget says in conclusion: "If I were to count the number of preventible calamities in surgery that I have known, I should find the majority of them due to the oversight of personal defects in the patients operated on; defects in the habits, the constitution, or the previous diseases, which ought to have been ascertained before the risk of the operation was incurred."

Another most excellent piece of advice is conveyed in the following language: "When you have decided on an operation, never make light of it. Never talk to the patient flippantly about its being what is called 'nothing,' a mere snip, a mere cut, a mere this or that. . . . You need not alarm the patient; you may say that the risk of an operation is not greater than that which he would incur for much less sufficient motives. . . . So you may fairly guard yourselves, and give your patients a just measure of warning, by saying that the risk of a proposed operation is not greater than the risk of this or that thing which they willingly do for amusement. But unless you are prepared to say that the risk is not greater than ought to be incurred for the good which may be expected to follow, you ought not to do the operation at all."

Upon the amount of good likely to follow an operation, Sir James Paget says: "The surgeon alone can in most cases estimate it. In most cases, therefore, we must take the whole responsibility of operations, for it is only on our statement that patients can rely in judging whether they should submit or not, and most of them, even when they have our statements before them, are quite incapable of clearly and soundly judging. But there is a class of operations, in what I have called decorative surgery, in which we may justly put upon patients a much larger share of responsibility than they ought to bear when the question is one of life or death. When the people want, not to be cured of absolute deformity, which hinders their success or comfort in life, but to have this or that done of which it does not matter whether it is done or not, except for some personal vanity, let them understand that the operation is not altogether free from risk, and then let them take the whole responsibility of the matter."

Under the second division of the subject, Sir James Paget speaks as follows: "Supposing, now, an operation to be decided on, first, don't be too ready to operate in your own houses or in your own rooms." After detailing the disastrous consequences of the removal, in his own room, of a simple encysted tumor from the back of a wealthy London merchant, by a surgeon of distinction, in the beginning of his professional career, the doctor goes on to say: "You may do an operation there with all dexterity and care, but you do not know what the patient will do afterwards." Especially let me advise you not to sound a patient for the first time, or to pass a catheter in a man of questional general health for the first time, in your own room.

Another judicious precaution is put in the following words: "Do not operate upon even small inflamed parts. * * * A man will bear a little tumor or a small cyst, or a small pile, so long as it is not inflamed; but when it inflames it teazes him, and he asks to have it removed with all speed. Don't do it. The risks of operating on an inflamed part are manifold, and much greater than the risks of operating on one that is quiet."

He then relates the fatal issue of an operation by himself upon a simple encysted tumor upon the abdomen, which was acutely inflamed at the time of the operation, as illustrating the wisdom of observing this precaution.

"For another rule, always look carefully to the condition of the room or the house in which your patient is living, and set aside, so far as you possibly can, all the risks that may thus be incurred. Look to the sanitary arrangements about the man." As an illustration of the wisdom of observing this rule, the author gives the details of a case of phymosis, upon which he operated by simple division of the prepuce, and which was followed by sloughing of the integuments, over two-thirds of the penis, and very nearly the whole of the scrotum. On looking about for the cause of this untoward result, he found that an article of furniture which he had before supposed to be a book-case, was a water-closet, the offensive smell from which had poisoned the atmosphere of the patient's apartment.

Another most salutary caution is put as follows: "Never do an operation if you can cure the patient by any reasonable medical or other means. There are a considerable number of operations done for cases that should not be operated on at all; and these are amongst the very class in which the mortality of minor operations comes."

"Then, for another rule," says the author, "If a disease can be cured by a bloodless operation as well as by one with cutting, choose the bloodless. This may be done in many instances than you are apt to think." Cysts of the scalp are given as an illustration of the cases in which removal by the knife is unnecessary, all cysts being removable with such greater safety by caustics. The ligature of hemorrhoids is another example given of the cases that sometimes prove fatal when caustics would have served as useful a purpose, and been free from the risks of the ligature. So with cancerous warts and ulcers that occur about the face.

Another rule: "Be quite clear about carrying out carefully the last stages of all operations. I suspect that everybody in operating, when he has passed through the sort of mental tension in which he performs the most difficult part of what he has to do, when his attention has been completely occupied in some difficult task to be achieved, next feels his mind relaxed, his attention less keen, less ready for exercise than it was before. Be sure that these are times of danger to your patient. As soon as the attention ceases to be as keen as possible, you are in risk of doing some mischief."

"One more rule I will give you: Look carefully to your apparatus. I have no doubt that you will look very carefully to the edges of your knives and your saws, and all things that are mighty to handle; but look to the plaster, look to the ligatures and the sutures, and all things which are commonly called minor. When I have seen Sir William Ferguson and Mr. Spencer Wells operate, I have never known which to admire most; the complete knowledge of the thing to be done, the skill of hand, or the exceeding care with which all the apparatus is adjusted and prepared beforehand. The most perfect plaster, the most perfect silk, not one trivial thing left short of the most complete perfection it is capable of. I have no doubt that the final success of their operations has been due just as much to these smaller things as to those greater things of which they are masters. In contrast with their work, I have seen operations performed with great skill, and a piece of bad plaster or bad silk, or something left at home, has put the patient's life in danger. Not long ago, I remember, a patient had secondary hemorrhage after an operation, and the reason was that the sticking plaster was bad. One of the things that was to control the hemorrhage was pressure by plaster; the plaster slipped, and the patient ultimately died of hemorrhage. Many an operation has been spoiled by bad silk, or bad needles, or bad something that was thought too trivial for care. Surgery could supply only too many illustrations of the wise proverb against those that despise small things."

The concluding clauses of this lecture contain such valuable counsel, especially to the young surgeon, that they are well worthy of being respected. The author says: "There is but one thing that I am afraid of in telling you the risks and dangers that I have met with, and that is that you may overestimate the probabilities of them, and be afraid of the responsibility which you must undertake. Well, after all, this incurring of responsibility is decided rather by temper than by knowledge. There are some people who are ready for anything; some that under difficulties shirk all they can. But of this I am quite sure, and you will see it proved, not only in surgery, but in very calling, the men who are most ready to take responsibilities and to bear them lightly, are those who can best estimate beforehand what are the risks and difficulties they incur; the men who, knowing what is to come, can therefore face it most bravely and with most success.

"Therefore study fairly and fully, beforehand, all the things that may occur to you in an operation and after it. Make yourselves, as far as you can, masters of each case, and generally masters of your profession, and then you will neither be afraid of your responsibilities nor ashamed of your failures."

